# Diffusion-Based Protein Structure Design: Geometric Modelling, Validation Strategies, and Thermodynamic Challenges

**DOI:** 10.3390/ijms27167151

**Published:** 2026-08-10

**Authors:** Wenran Li, Xavier Cadet, David Medina-Ortiz, Mehdi D. Davari, Ramanathan Sowdhamini, Miloud Bessafi, Cedric Damour, Yu Li, Alain Miranville, Alexandre G. de Brevern, Frederic Cadet

**Affiliations:** 1 Université Paris Cité & Université de la Réunion, INSERM, EFS, BIGR U1134, DSIMB Bioinformatics Team, 75015 Paris, France; 2 Thayer School of Engineering, Dartmouth College, Hanover, NH 03755, USA; 3 Departamento de Ingeniería en Computación, Universidad de Magallanes, Avenida Bulnes, Punta Arenas 01855, Chile; 4 Department of Bioorganic Chemistry, Leibniz Institute of Plant Biochemistry, 06120 Halle, Germany; 5 National Centre for Biological Science, TIFR, Bangalore 560065, India; 6 Molecular Biophysics Unit, Indian Institute of Science, Bangalore 560012, India; 7 Institute of Bioinformatics and Applied Biotechnology, Bangalore 560066, India; 8 ENERGYLab, EA 4079, Faculté des Sciences et Technologies, Université de La Reunion, 97490 Saint-Denis, Francecedric.damour@univ-reunion.fr (C.D.); 9 School of Information Science and Technology, Beijing Institute of Artificial Intelligence, Beijing 102206, China; 10 Laboratoire de Mathématiques Appliquées, University Le Havre Normandie, 76600 Le Havre, France; 11 PEACCEL, AI for Biologics, 75013 Paris, France

**Keywords:** diffusion models, protein structure generation, protein engineering, geometric deep learning, SE(3)-equivariance, structural validation, thermodynamic stability

## Abstract

Although deep learning has transformed protein structure prediction, the controlled generation of functional and experimentally tractable protein structures remains a major challenge in structural bioinformatics. Diffusion models offer a versatile approach to generating protein backbones, motif-conditioned scaffolds, all-atom structures and biomolecular interaction geometries, while accommodating explicit structural and functional constraints. This review focuses on coordinate- and residue-frame-based diffusion approaches for generating protein structures, paying particular attention to geometric equivariance, conditioning strategies, all-atom modelling and interaction-aware design. We compare representative methods derived from RoseTTAFold, frame-diffusion architectures, and oriented-residue-cloud representations according to their molecular representation, generative objective, and validation strategy. We examine the criteria used to evaluate generated proteins, such as stereochemical quality, structural consistency, designability, novelty, diversity, computational efficiency, and experimental performance. Particular attention is given to the distinction between learned structural distributions and condition-dependent thermodynamic ensembles. Future progress will depend on the integration of generative models with molecular mechanics, conformational sampling, uncertainty estimation, free-energy methods, and experimental design–build–test–learn cycles. Within this framework, diffusion models offer candidate generation and constraint satisfaction capabilities within broader protein engineering workflows.

## 1. Introduction

The objective of protein engineering is to identify amino-acid sequences that adopt specified three-dimensional structures while satisfying functional, thermodynamic, kinetic and developability constraints [1]. The inverse problem is inherently multi-objective because sequence, structure, conformational dynamics, stability, molecular recognition, and biological function are strongly interconnected across a complex, high-dimensional energy landscape [2]. Notwithstanding the existence of a target fold that has been predetermined, the accessible sequence space grows combinatorially with chain length [3]. This renders an exhaustive exploration of the sequence space an impractical undertaking. Classical approaches, encompassing rational design, directed evolution and energy-based optimisation, have yielded substantial success. Nevertheless, their performance continues to be constrained by conformational sampling, imperfect scoring functions and experimental throughput [4]. The current transformation is therefore not only a matter of better structure prediction. It is a broader shift from the deterministic modelling of individual structures toward the generative exploration of distributions over sequences, folds, complexes and functional constraints.

Deep learning changed this landscape by making structural information more accessible at scale. AlphaFold2 established that end-to-end neural architectures can predict many protein structures with high accuracy from sequence and evolutionary information [5]. RoseTTAFold and related three-track architectures further demonstrated the value of coupled sequence, distance and coordinate representations [6]. AlphaFold3 and RoseTTAFold All-Atom extended the scope of prediction toward molecular complexes and non-protein components, including ligands, nucleic acids, ions and covalent modifications [7,8]. These predictors constitute a significant element of contemporary design pipelines. However, structure prediction in isolation does not resolve the inverse design problem. A predictor estimates the structure or interaction geometry associated with a candidate sequence or molecular system. Conversely, a design method must generate candidates that satisfy predefined structural, functional or physicochemical constraints [9]. In practice, prediction and generation are increasingly integrated, as generative models propose sequences or structures while structure predictors, inverse-folding models, and property estimators provide iterative filtering or guidance before experimental testing.

Diffusion models have become especially influential in this context. In their standard formulation, a forward process progressively perturbs data according to a predefined noise schedule. In turn, a learned reverse process iteratively transforms a sample from a simple prior distribution into an approximation of the training-data distribution [10,11]. This approach has several properties that are attractive for protein engineering [12]. First, diffusion can be conditioned on motifs, binding sites, symmetry, secondary-structure patterns or sequence embeddings. Second, denoising can be implemented in coordinate space, latent space, graph space or sequence space. Third, the sampling trajectory provides natural entry points for guidance, partial diffusion and constraint enforcement. The properties outlined herein have enabled the development of diffusion-based methods, which have in turn been employed to address several distinct molecular-design tasks. These include the generation of protein sequences, inverse folding, the generation of backbones, motif scaffolding, the generation of all-atom complexes and conformational ensemble sampling [13,14,15,16,17]. These applications should nevertheless be distinguished from one another because they operate on different molecular representations, use different conditioning strategies and require different validation criteria.

The biological relevance of diffusion-based design depends on how molecular structure is represented. Protein backbones are not unstructured point clouds; they are ordered polymers with stereochemical constraints, local frames and long-range packing interactions [18]. Many successful structure-generation models therefore use equivariant architectures that preserve the behaviour of coordinates under rigid transformations. Representative examples include SchNet, which introduced continuous-filter convolutions for atomistic systems; Tensor Field Networks (TFN) and SE(3)-Transformer, which perform equivariant message passing over 3D point clouds; and protein-specific frameworks such as RoseTTAFold, AlphaFold2, and RFdiffusion, which incorporate SE(3)-equivariant operations to maintain consistent residue-frame geometry during structure prediction and generation [5,18,19,20]. In protein design, SE(3)-equivariance is commonly employed because global rotations and translations should not affect the identity of the design problem. However, reflections can invert molecular chirality. Chirality can also be treated as an explicit design variable rather than merely as a constraint to be preserved. The IDeAS framework has demonstrated the automated inverse design of heterochiral protein folds that incorporate both L- and D-amino acids. This shows that residue stereochemistry can be introduced explicitly into the protein design space. While IDeAS is not a diffusion model, it shows how stereochemical configuration can be incorporated as a molecular design degree of freedom [21]. SE(3)-equivariant models therefore provide an appropriate inductive bias for coordinate generation, ensuring transformation-consistent predictions under proper rotations and translations. E(3)-equivariant formulations also include reflections and consequently require the explicit consideration of molecular handedness in chiral systems [22]. While these geometric properties improve coordinate consistency and data efficiency, the chemical and stereochemical interpretation of generated structures requires separate assessment [23].

Recent applications illustrate how diffusion-based protein design has moved from methodological demonstration to experimentally oriented protein engineering. RFdiffusion-derived strategies have been used to design high-affinity binders against bioactive helical peptides, showing that denoising trajectories can generate protein scaffolds around flexible peptide targets and recover experimentally validated binding modes [24]. This example illustrates how diffusion-based design extends structure generation beyond unconditional fold sampling and explicitly links generative modelling to molecular recognition. In parallel, RFdiffusion All-Atom expanded the design space toward heterogeneous biomolecular systems by generating protein structures around small molecules and other non-protein components [8]. Such all-atom approaches are particularly relevant for protein engineering because they connect backbone generation, side-chain placement, ligand recognition and local stereochemical constraints within a single design problem.

Although several reviews have described generative modelling for proteins, the relationships between molecular representation, geometric conditioning, physical consistency, computational validation and experimental evidence are still not fully understood [22,25,26,27]. This review therefore focuses on diffusion-based methods for generating three-dimensional protein structures. The approaches are examined according to the structural representation employed, the constraints imposed during sampling, and the validation procedures used to assess the generated candidates. First, the main diffusion formulations relevant to coordinate- and residue-frame-based generation are introduced, together with the role of geometric equivariance. Representative methods are then organised by their structural design objectives, which include unconditional backbone generation, motif scaffolding, programmable structure generation, all-atom modelling, and interaction-aware design. Sequence-only diffusion, general protein language models, inverse-folding methods and molecular docking approaches are considered only when they contribute directly to structural generation workflows and are not reviewed comprehensively. Validation strategies are then examined at the stereochemical, structural, functional, and experimental levels. Finally, the difference between a generative distribution learned from data and a physical equilibrium distribution is discussed, as well as the potential contributions of molecular mechanics, conformational sampling, free-energy calculations and experimental feedback to thermodynamics-informed protein engineering.

This review makes three key contributions. Firstly, it establishes a conceptual framework linking diffusion formulations to the coordinate and residue-frame representations used in generating protein structures. Secondly, it provides a critical, task-oriented comparison of representative backbone, motif-conditioned, all-atom, and interaction-aware diffusion models. Particular attention is paid to conditioning mechanisms, evaluation protocols, and available experimental evidence. Thirdly, it distinguishes between geometric plausibility, structural self-consistency, predicted designability, thermodynamic stability and biological function. Based on these, this review identifies the methodological developments required to integrate physically grounded constraints, uncertainty estimation, conformational sampling and experimental feedback into diffusion-based protein engineering workflows.

## 2. Theoretical Background

The principal formulations underlying diffusion-based protein generation are introduced in this section. This section first summarises denoising diffusion probabilistic models (DDPMs) and continuous-time score-based generative models, and then introduces geometric invariance and equivariance for coordinate-based molecular generation [10,28]. The focus of this study is the relationship between the mathematical representation of molecular structures and the transformation properties imposed on the generative model.

### 2.1. Diffusion Models

**Figure 1:**
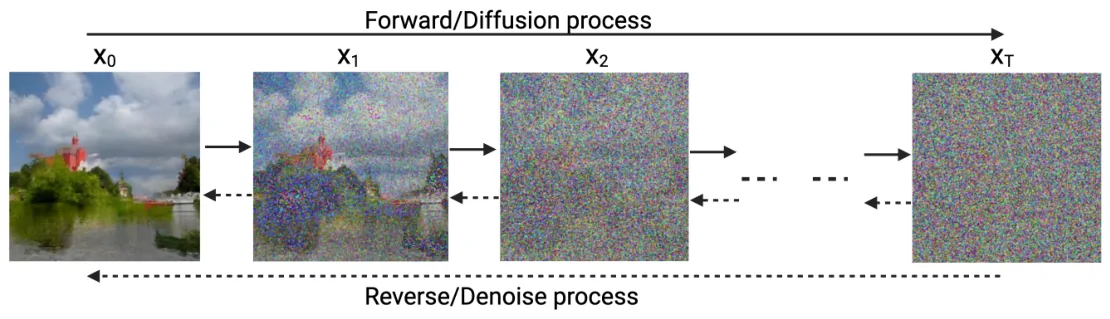
Illustration of forward and reverse diffusion processes using image generation as an example. During the forward process, the image is progressively corrupted by Gaussian noise until it approaches an isotropic Gaussian distribution. The reverse process is a denoising process, and progressively denoises the noisy states to recover a clear image. The solid right-pointing arrows indicate successive forward diffusion steps, in which noise is progressively added from $x_{0}$ toward $x_{T}$. The dashed left-pointing arrows indicate successive reverse denoising steps, in which the model progressively reconstructs less noisy states from $x_{T}$ toward $x_{0}$. The long dashed line at the bottom summarizes the overall reverse trajectory from the final noisy state $x_{T}$ to the initial clean state $x_{0}$, rather than representing an individual denoising step.

Diffusion models define a generative process through two complementary components: a forward noising process and a learned reverse denoising process, as shown in Figure 1. Let $x_{0}∼q_{\mathrm{data}}\left(x\right)$ denote a data sample, which may correspond to an amino-acid sequence, a backbone-coordinate representation, a residue-frame representation, or a latent molecular representation. The forward process progressively perturbs $x_{0}$ according to a predefined noise schedule until the distribution approaches a simple prior, typically an isotropic Gaussian distribution. The reverse process is then learned by a parameterised model that gradually transforms samples from this prior back toward the data distribution. The notation used throughout the theoretical background is summarised in Table 1.

In the discrete-time formulation of denoising diffusion probabilistic models (DDPMs), the forward process is defined by a Markov chain

(1) $$\;q\left(x_{t}|x_{t-1}\right)=N\left(x_{t};\sqrt{1-\beta_{t}}\;x_{t-1},\beta_{t}I\right),\;t=1,\ldots ,T,$$

where $\beta_{t}\in \left(0,1\right)$ is a predefined variance schedule and *T* is the total number of diffusion steps. It is standard to define

(2) $$\alpha_{t}=1-\beta_{t},\;{\bar{\alpha }}_{t}=\prod_{s=1}^{t}\alpha_{s}.$$

With this notation, the marginal distribution of $x_{t}$ conditioned on $x_{0}$ can be written in closed form as

(3) $$q\left(x_{t}|x_{0}\right)=N\left(x_{t};\sqrt{{\bar{\alpha }}_{t}}\;x_{0},\left(1-{\bar{\alpha }}_{t}\right)I\right).$$

Equivalently, $x_{t}$ can be sampled directly from $x_{0}$ by

(4) $$x_{t}=\sqrt{{\bar{\alpha }}_{t}}\;x_{0}+\sqrt{1-{\bar{\alpha }}_{t}}\;\epsilon ,\;\epsilon ∼N\left(0,I\right).$$

The reverse process starts from a sample $x_{T}$ drawn from a simple prior, usually $x_{T}∼N\left(0,I\right)$, and learns a sequence of denoising transitions:

(5) $$p_{\theta }\left(x_{0:T}\right)=p\left(x_{T}\right)\prod_{t=1}^{T}p_{\theta }\left(x_{t-1}|x_{t}\right).$$

Each reverse transition is commonly parameterised as a Gaussian distribution:

(6) $$p_{\theta }\left(x_{t-1}|x_{t}\right)=N\left(x_{t-1};\mu_{\theta }\left(x_{t},t\right),\Sigma_{\theta }\left(x_{t},t\right)\right),$$

where $\mu_{\theta }\left(x_{t},t\right)$ is the learned reverse mean and $\Sigma_{\theta }\left(x_{t},t\right)$ denotes the reverse covariance. In the commonly used noise-prediction parameterisation, a neural network $\epsilon_{\theta }\left(x_{t},t\right)$ is trained to predict the Gaussian noise ϵ added in the forward process. The reverse mean can then be expressed as

(7) $$\mu_{\theta }\left(x_{t},t\right)=\frac{1}{\sqrt{\alpha_{t}}}\left(x_{t}-\frac{\beta_{t}}{\sqrt{1-{\bar{\alpha }}_{t}}}\epsilon_{\theta }\left(x_{t},t\right)\right).$$

The simplified DDPM training objective is therefore

(8) $$L_{\mathrm{DDPM}}\left(\theta \right)=E_{x_{0},t,\epsilon }\left[{∥\epsilon -\epsilon_{\theta }\left(\sqrt{{\bar{\alpha }}_{t}}x_{0}+\sqrt{1-{\bar{\alpha }}_{t}}\epsilon ,t\right)∥}_{2}^{2}\right],$$

where $x_{0}∼q_{\mathrm{data}}\left(x\right)$, *t* is sampled from the diffusion time steps, and ϵ∼N(0,I). This objective trains the model to recover the noise used to construct $x_{t}$, but does not directly predict the covariance of the reverse transition.

Diffusion models can also be formulated in continuous time through stochastic differential equations (SDEs). A general forward SDE can be written as

(9) $$dx=f\left(x,t\right)\;dt+g\left(t\right)\;d\omega_{t},$$

where f(x,t) is the drift term, g(t) is the diffusion coefficient, and $\omega_{t}$ is a standard Wiener process. Let $p_{t}\left(x\right)$ denote the marginal distribution of $x_{t}$ at time *t*. The corresponding reverse-time SDE is

(10) $$dx=\left[f\left(x,t\right)-g{\left(t\right)}^{2}\nabla_{x}\log p_{t}\left(x\right)\right]dt+g\left(t\right)\;d{\bar{w}}_{t},$$

where ${\bar{w}}_{t}$ is the reverse-time Wiener process. The associated probability-flow ordinary differential equation (ODE), which has the same marginal distributions as the SDE, is

(11) $$dx=\left[f\left(x,t\right)-\frac{1}{2}g{\left(t\right)}^{2}\nabla_{x}\log p_{t}\left(x\right)\right]dt.$$

Score-based generative models learn the time-dependent score function

(12) $$\nabla_{x}\log p_{t}\left(x\right),$$

which is the gradient of the log-density of the perturbed data distribution at time *t*. A score network $s_{\theta }\left(x,t\right)$ is trained to approximate this quantity, often through denoising score matching:

(13) $$L_{\mathrm{score}}\left(\theta \right)=E_{t,x_{0},x_{t}}\left[\lambda \left(t\right){∥s_{\theta }\left(x_{t},t\right)-\nabla_{x_{t}}\log q_{t}\left(x_{t}|x_{0}\right)∥}^{2}\right],$$

where $q_{t}\left(x_{t}|x_{0}\right)$ is the conditional perturbation kernel and λ(t) is a time-dependent weighting function.

Discrete-time DDPMs and continuous-time score-based models are closely related. The DDPM formulation is often convenient for describing finite-step denoising algorithms, whereas the SDE formulation provides a unified view of diffusion, reverse-time sampling and probability-flow dynamics. In protein design, these formulations can be applied to different molecular representations, including sequences, backbone coordinates, residue-local frames, all-atom coordinates and latent embeddings [29]. The choice of representation determines which molecular constraints can be imposed during sampling and what type of validation is required after generation.

### 2.2. Practical Considerations for Protein Design

Although diffusion models offer a flexible probabilistic framework, their practical application hinges on the combination of molecular representations, conditioning variables, and downstream evaluation procedures. Sequence-based models operate on amino-acid identities, whereas coordinate-based and all-atom approaches generate backbone geometry, side-chain conformations, and heterogeneous biomolecular assemblies directly. The selected representation determines whether conditioning can be imposed on motifs, interfaces, catalytic residues, ligands, symmetry, or other design constraints.

Current protein-engineering workflows therefore combine diffusion models with inverse-folding methods, structure predictors, property estimators, structural refinement, and experimental screening. The following sections are organised according to these molecular representations and practical design objectives, rather than solely according to the underlying mathematical formulation.

## 3. Structural Diffusion Models for Protein Engineering

Recent diffusion approaches have evolved from unconditional backbone generation to include motif-conditioned scaffolding, programmable generation, all-atom design, and heterogeneous biomolecular modelling. Table 2 summarises representative methods according to their molecular representation, design objective, and principal application domain. The following comparison is organised around practical questions that arise when selecting a method, such as whether the task requires an unconstrained backbone, the preservation of one or more structural motifs, the atom-level control of molecular interactions, joint sequence-structure generation or the sampling of alternative conformational states. For each model family, we consider the architectural formulation together with representative applications and the type of conditioning supported, as well as the principal limitations of the available validation evidence.

SE(3)-equivariant models ensure that coordinate updates transform consistently under proper rotations and translations. For protein backbones, residue-local $N—C_{\alpha }—C$ frames provide a natural representation of residue position and orientation while retaining molecular handedness under these transformations. This removes dependence on an arbitrary global reference frame and facilitates the generation of transformation-consistent protein geometries. A concise mathematical description of SE(3) is provided in Appendix B.

**Figure 2:**
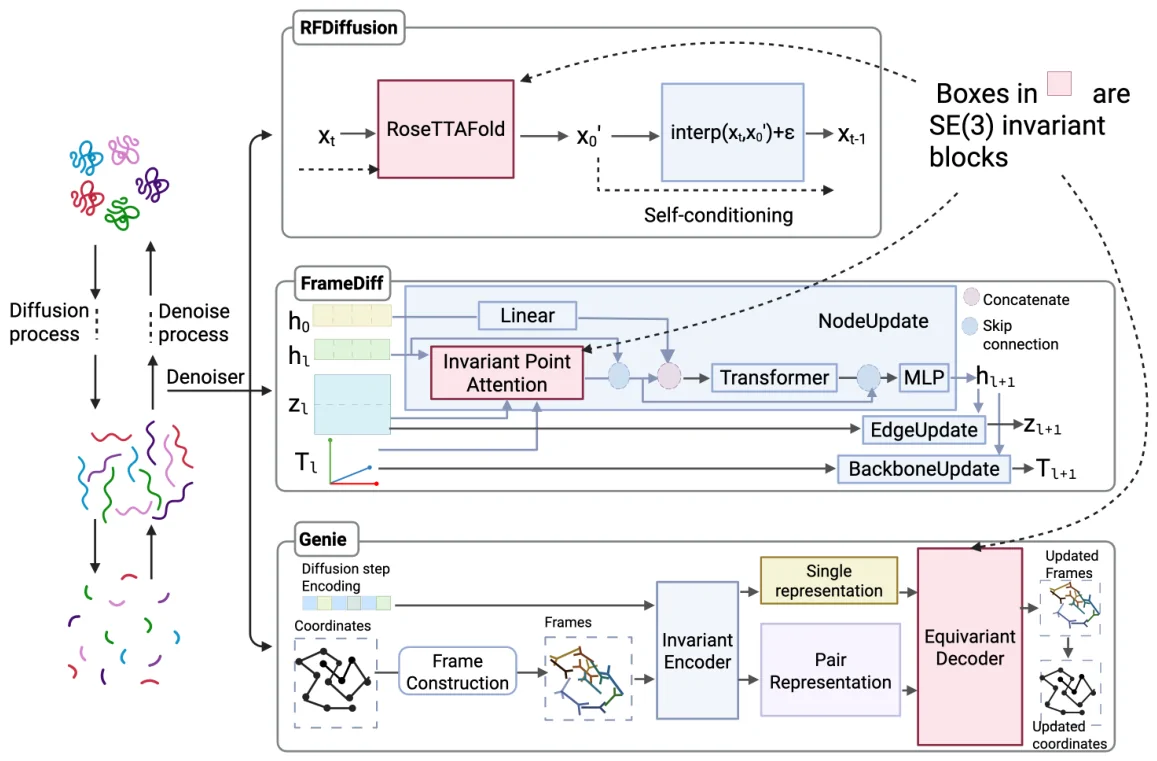
SE(3)—equivariant diffusion architectures for protein—backbone generation. RFdiffusion, FrameDiff and Genie employ different denoising architectures based on RoseTTAFold representations, residue−frame diffusion with Invariant Point Attention and oriented residue cloud modelling, respectively. Solid arrows indicate the principal flow of representations, coordinates, or updated states through each denoising architecture. Dashed arrows within the architectures indicate auxiliary connections, including self-conditioning and skip or residual information flow. Dashed lines extending across modules are explanatory links connecting the highlighted components to the annotation of SE(3)-invariant or equivariant operations, rather than additional computational pathways. The pink boxes indicate the SE(3)-invariant or equivariant modules involved in processing or updating geometric information in a manner consistent with global rotations and translations.

SE(3) equivariance has become one of the fundamental design principles for physically plausible protein structure generation. Each residue is represented by its local $N—C_{\alpha }—C$ coordinate frame, which provides a rotation- and translation-consistent representation for backbone generation. Therefore, most recent diffusion-based protein structure generators adopt SE(3)-equivariant neural networks as the denoising backbone. As illustrated in Figure 2, current approaches can be broadly categorised into three representative families: the RFDiffusion family, the FrameDiff family, and the Genie family.

RFdiffusion employs RoseTTAFold−derived structural representations for iterative backbone denoising [13]. In the process of unconditional generation, the method samples de novo protein backbones without the requirement of a predefined fold. In conditional applications, selected residues, structural motifs or target−contact geometries can be retained while the surrounding scaffold is generated. This formulation has enabled the execution of motif−scaffolding tasks, in which catalytic or interaction−relevant structural elements must be positioned within a compatible protein framework. Furthermore, RFdiffusion−derived workflows have been applied to the design of high−affinity binders targeting bioactive helical peptides and intrinsically disordered proteins [24,30]. These applications illustrate the suitability of RFdiffusion when a target geometry or functional motif is available, although the generated backbone must generally be followed by sequence design, structure−prediction−based filtering and experimental testing.

The RFdiffusion family has progressively extended backbone generation towards atom−level molecular design. RoseTTAFold All−Atom is chiefly a structure-prediction architecture for heterogeneous biomolecular assemblies [8], whereas RFdiffusionAA adapts this representation to generate protein structures around specified non−protein components. The reported applications encompass the design of proteins surrounding digoxigenin, heme and optically active bilin molecules, thereby demonstrating conditioning on small molecules and cofactors with distinct chemical geometries. RFdiffusion2 extends this strategy by accepting atom−level descriptions of functional sites, rendering it particularly suitable for active−site scaffolding, in which the precise reproduction of catalytic atoms or ligand−contact geometries is paramount [31]. RFdiffusion3 introduces a natively all-atom generative formulation that allows a more direct specification of hydrogen bonds, ligand contacts and interactions with nucleic acids, while reducing the computational cost relative to RFdiffusion2 [32]. It is therefore evident that these methods are most relevant when the design objective is defined at atomic rather than backbone resolution. However, the success of the application process is contingent on factors such as compatible sequence assignment, efficient side−chain packing, effective protonation−state treatment, and experimental verification of the intended interaction.

FrameDiff represents a protein backbone as a sequence of residue-local rigid frames. It defines diffusion directly over their rotational and translational components [14]. This representation is particularly well-suited to unconditional backbone generation, as it treats residue position and orientation in a unified manner during the denoising process. Furthermore, it provides a natural basis for conditional reconstruction tasks. FrameDiPT extends the framework to protein-structure inpainting, in which missing residues or segments are generated while the observed structural context remains fixed [33]. This approach may be useful for loop reconstruction, domain completion or the generation of structural regions connecting predefined components. TDS provides conditional sampling for motif-scaffolding problems without requiring a separately retrained model [34], whereas VFN-Diff replaces Invariant Point Attention with Vector Field Networks and reports improved designability and diversity in computational benchmarks [35].

These methods are most effective when the design problem can be expressed using backbone frames, fixed structural contexts, or partial coordinates. The primary constraint pertains to the reliance of performance comparisons on computational self-consistency, novelty and diversity metrics. It is evident that the generation of backbones still necessitates downstream sequence design and independent assessment of foldability, stability, and function.

Genie employs a computationally straightforward coordinate-noising process in the forward direction and reconstructs oriented residue frames during denoising [15]. In its original formulation, the method is primarily applicable to the unconditional generation of diverse and designable monomeric backbones. The Genie2 framework extends this methodology to motif scaffolding, with particular application to multi-motif design, where the relative positions and orientations of multiple motifs do not require prior specification [36]. Representative use cases include the generation of enzyme-like scaffolds containing multiple substrate-contacting elements and proteins designed to present several interaction motifs within a common structure. Genie3 introduces all-atom representations and sampling procedures, extending the family towards long-monomer generation, motif scaffolding and binder design [37].

The Genie family is therefore particularly relevant when structural exploration or multi-motif arrangement is prioritised over the strict preservation of a fully predefined scaffold. The improvements that have been reported are primarily based on computational benchmarks. Nevertheless, a direct comparison with RFdiffusion or FrameDiff remains sensitive to differences in training data, sequence-design procedures, sampling budgets and evaluator choice.

Chroma tackles design issues that call for programmable combinations of structural and semantic constraints, as opposed to a solitary fixed motif [16]. The employment of user-defined energy functions has been demonstrated to facilitate the generation of structures that exhibit specific symmetries, global shapes, retained substructures, or semantic attributes. These attributes are often associated with a particular protein class or a specific design objective. Representative applications of this technology therefore include the generation of symmetric assemblies, shape-constrained proteins and structures containing prescribed subcomponents. This flexibility is advantageous when the design specification cannot be reduced to conventional motif scaffolding. Nevertheless, the provision of robust user-defined guidance has the potential to modify the sampled distribution, and the resultant candidates must undergo the same independent structural and experimental validation as other conditionally generated proteins.

Several specialised approaches extend diffusion beyond the generation of backbones only. ProteinGenerator couples sequence-space diffusion to structural generation, making it applicable to joint sequence-structure and multistate design problems, where sequence and conformation must be optimised together [23]. AlphaFold3 employs a diffusion module for conditional all-atom coordinate generation, but its primary function is to predict heterogeneous biomolecular complexes from specified molecular components rather than to design proteins de novo [7]. However, within a design pipeline, it can be used to evaluate candidate protein–protein, protein–ligand, and protein–nucleic acid complexes. DiffDock specialises in ligand-pose generation and molecular docking, falling outside the central scope of backbone design [38].

Mac-Diff addresses a different application, learning distributions of protein conformations from molecular dynamics trajectories [39]. It can generate alternative conformational states and explore structural heterogeneity rather than a single static backbone. However, the resulting samples should be interpreted as learned conformational distributions rather than as equilibrium ensembles with physically calibrated populations or kinetic connectivity. Protpardelle jointly models backbone geometry, amino-acid sequence and side-chain states, making it highly relevant to the distinction between backbone-only generation and all-atom generative design [39].

## 4. Evaluation Metrics, Benchmark Frameworks and Experimental Validation

Protein-generative models should be evaluated using a set of complementary criteria, chosen based on the intended design task. This section distinguishes between local stereochemical quality, sequence-structure self-consistency, novelty and diversity, satisfaction of task-specific constraints, computational efficiency, and experimental performance. As benchmark outcomes depend on datasets, sampling budgets, evaluators and filtering protocols, meaningful comparisons require the transparent reporting of each component of the evaluation pipeline. Table 3 summarises the major evaluation metrics and benchmark frameworks discussed in this section, together with the validation layers they assess and their principal limitations.

MolProbity is a widely utilised tool for the evaluation of the local stereochemical quality of protein structures that have been either experimentally determined or computationally generated [40]. The analysis encompasses all-atom steric clashes, Ramachandran outliers, side-chain rotamer quality, and selected geometric deviations. These criteria are useful for identifying locally unrealistic conformations, but they do not establish global fold stability, correct oligomerisation, favourable solvation or biological function. MolProbity should therefore be interpreted as a stereochemical validation tool, not as a comprehensive measure of physical plausibility. The following discussion examines these validation layers in greater detail, beginning with local stereochemical assessment and progressing towards benchmark-level and experimental evidence.

PDB-Struct is a method of evaluating structure-based protein design that employs complementary measures of structural self-consistency and experimentally annotated stability [41]. The structural similarity between a target backbone and a predicted structure can be quantified using metrics such as TM-score. The term ’predicted local distance difference test’ (pLDDT) refers to a per−residue confidence score that is estimated by a structure−prediction model. TM-score and pLDDT should not be interpreted equivalently. TM-score quantifies global structural similarity between two aligned structures, whereas pLDDT is a per−residue confidence estimate produced by a structure-prediction model. A high pLDDT value indicates that the predictor is confident in the local geometry, but it does not provide direct evidence that the sequence is thermodynamically stable, experimentally foldable, or functionally active. In PDB−Struct, thermodynamic stability is instead evaluated using experimentally annotated mutagenesis datasets. For the stability metric, PDB−Struct reports the Spearman correlation between model pseudo−log−likelihood and experimentally measured stability. The stability score is defined as

$$R\left(\theta ,D\right)=\rho_{s}\left({\left\{L_{\theta }\left(S^{\left(i\right)}|X_{\mathrm{template}}^{\left(i\right)}\right)\right\}}_{i=1}^{n},{\left\{G^{\left(i\right)}\right\}}_{i=1}^{n}\right),$$

where $\rho_{s}$ is Spearman’s correlation, θ is the design model, L is the pseudo-log-likelihood function and $D={\left\{\left(X_{\mathrm{template}}^{\left(i\right)},S^{\left(i\right)},G^{\left(i\right)}\right)\right\}}_{i=1}^{n}$ is the evaluation dataset, with $X_{template}$ the template structure, $S^{\left(i\right)}$ the *i*-th sequence and $G^{\left(i\right)}$ the stability score corresponding to the *i*-th sequence. The evaluation dataset consists of experimental mutagenesis datasets for de novo protein designs containing experimentally measured stability annotations. PDB-struct suggests that encoder–decoder methods generally outperform structure-prediction-based methods in terms of refoldability, recovery, and stability metrics.

Scaffold-Lab is a recent approach and evaluates protein generators on tasks such as unconditional backbone generation and motif scaffolding using several complementary dimensions, including designability, novelty, diversity, computational efficiency and structural quality [42]. The designability of a generated backbone is typically assessed through a self-consistency procedure in which sequences are designed for that backbone and subsequently evaluated using an independent structure−prediction model. The quantification of structural recovery can then be conducted through the use of RMSD, TM−score, or analogous metrics. RMSD and pLDDT should not be treated as interchangeable. RMSD measures the mean geometric deviation between corresponding atoms after structural superposition. Structural novelty is frequently estimated from the maximum similarity between a generated backbone and entries in a reference database, for example, using the highest TM−score obtained against the Protein Data Bank. Lower nearest−neighbour similarity can indicate greater structural novelty, but the result depends on the database release, chain-length distribution, structural-search method and alignment procedure. Moreover, novelty should not be interpreted as evidence of designability or function because highly novel structures may also be physically unrealistic or difficult to stabilise experimentally. Diversity is the number of unique clusters divided by the total number of protein backbones in each group:

$$\mathrm{Diversity}=\frac{N_{\mathrm{unique}\;\mathrm{clusters}}}{N_{\mathrm{total}\;\mathrm{number}\;\mathrm{of}\;\mathrm{proteins}}}.$$

In general, a higher count of clusters reflects more heterogeneous topologies present within a subset of sampled protein backbones.

A recent example illustrating the careful interpretation of computational validation is the SALAD family of sparse denoising models proposed by Jendrusch and Korbel. In addition to demonstrating scalable backbone generation for proteins approaching 1000 residues and applications to motif scaffolding, structure editing and multistate design, the authors explicitly note that predictor-based designability metrics alone are insufficient and that additional experimental validation remains necessary. This provides an instructive example that computational ranking should not be interpreted as experimental evidence [43].

Melodia is a Python library for protein structural analysis and visualisation that includes descriptors derived from differential geometry and knot theory [44]. The use of such tools has the potential to enhance the evaluation of generated structures by characterising local backbone geometry, curvature, or topological complexity. However, Melodia does not itself serve as a benchmark for generative protein design, and therefore should be distinguished from evaluation frameworks that compare models across standardised datasets and tasks. The inclusion of such a term is justified only if the specific descriptors used to assess generated structures are clearly defined.

These evaluation criteria constitute a hierarchy rather than a single, composite definition of protein quality. The following should be reported separately: local geometry; sequence-structure recovery; distributional novelty; and task-specific constraint satisfaction. Where the design objective requires a physical or functional interpretation, these computational criteria should be supplemented with physically motivated calculations and experimental measurements. Interpretation of the results also requires controls for training-data leakage, evaluator dependence and candidate-selection bias.

The interpretation of benchmarks is rendered even more complex when generators and evaluators depend on related architectures, training databases or evolutionary information. It is therefore recommended that benchmark protocols report structure-level redundancy filters, temporal splits, evaluator independence, sampling budgets and the number of candidates retained after filtering. In order to reduce survivorship bias and publication bias, experimental failures should be reported wherever possible.

It is important to distinguish between experimental validation and computational ranking, and this distinction must be reported at several levels. The preliminary evaluation may encompass recombinant expression, solubility, and monodispersity. The validation of structures may be achieved through a variety of methods, including circular dichroism, nuclear magnetic resonance, crystallography, and cryo-electron microscopy. Depending on the objective of the design, functional validation may require measurements of binding affinity, specificity, catalytic activity or cellular response. The proportion of candidates that have been tested through experimentation, and the success rate at each validation stage, are essential for the comparison of generative methods. However, these statistics are currently reported inconsistently across studies.

## 5. Discussion

Diffusion models have expanded the scope of protein generation, moving from unconditional backbone sampling to motif scaffolding, binder design, and all-atom interaction modelling. The main methodological advantage of these models is the combination of flexible conditioning with geometric molecular representations, which allows structural constraints to be introduced throughout iterative denoising. However, their limitations are task-dependent: sampling can be computationally expensive, and performance can be sensitive to noise schedules and guidance parameters. Furthermore, their comparative advantages over autoregressive models, masked language models, flow-matching approaches, and energy-based optimisation remain incompletely established.

A second major trend is the reuse of structure-prediction architectures within generative workflows. For example, RFdiffusion adapts RoseTTAFold-derived representations for constrained backbone generation, whereas AlphaFold3 uses a diffusion module to predict coordinates for specified multicomponent systems. These examples illustrate the increasing convergence of prediction, generation, and downstream filtering while retaining distinct objectives and evaluation requirements. Therefore, the practical value of a method depends less on the presence of diffusion alone than on the correspondence between its molecular representation, conditioning scheme, sampling cost, and intended experimental application.

### 5.1. Limitations

Despite these remarkable achievements, several important challenges remain before diffusion models can become routine tools for practical protein engineering.

Firstly, the composition of training datasets imposes limitations on model generalisation. Structural databases demonstrate an overrepresentation of proteins that are experimentally tractable, stable and well-ordered, while membrane proteins, intrinsically disordered regions, transient assemblies, alternative conformational states and unsuccessful experimental constructs remain comparatively underrepresented. Sequence databases also contain significant imbalances with regard to taxonomy, family and annotation. Generative models may reproduce the structural and evolutionary biases of their training data and demonstrate reduced reliability for protein classes that are poorly represented. The extent of this limitation is contingent on factors such as dataset curation, redundancy reduction, training objectives and the use of predicted structures or synthetic data.

Secondly, protein dynamics and intrinsic disorder remain incompletely represented. A significant number of contemporary models yield a solitary structure or an assortment of independently sampled conformations. However, biological function may be contingent on heterogeneous ensembles, ligand-coupled transitions, allosteric communication and disorder-to-order processes. A collection of structurally diverse samples should not be automatically interpreted as an equilibrium ensemble, since the generated conformations may lack physically meaningful populations, kinetic connectivity, or transition pathways. Thus, ensemble-oriented diffusion models must be validated against molecular simulations, experimental observables, or independently estimated free-energy landscapes before thermodynamic or kinetic interpretations can be assigned.

Thirdly, the complexity of functional design remains significantly more challenging than that of geometric generation. Protein function cannot be adequately represented by a single annotation, since successful design may require binding affinity, molecular specificity, catalytic efficiency, substrate selectivity, allosteric response, cellular activity or compatibility with a particular biological environment. Functional labels are generally scarcer, more heterogeneous and more condition-dependent than structural data. Therefore, a generated protein may satisfy a structural constraint without achieving the intended biochemical activity or without avoiding competing interactions. Function-guided generation necessitates task-specific experimental measurements, precluding validation solely through predicted structural compatibility. Closing the gap between structural plausibility and biological functionality will require tighter integration between generative models, experimental data, and mechanistic biological knowledge.

Fourthly, current generative architectures do not fully encode the chemical and environmental context. Structures produced in residue-frame or Cartesian coordinate space may contain local steric clashes, strained torsions, inadequate side-chain packing, incorrect protonation states, or interactions that are incompatible with the solvent. While stereochemical restraints, molecular mechanics and energy-based guidance can mitigate some of these issues, their effectiveness hinges on the accuracy of force fields, protonation and solvent models, as well as the extent of conformational sampling. Therefore, these procedures should be reported as explicit components of the generation or refinement protocol, alongside their computational cost and candidate-acceptance criteria.

Fifthly, evaluation protocols remain insufficiently standardised. TM-score, RMSD, pLDDT, sequence recovery, novelty, diversity and self-consistency all quantify different properties and should therefore be reported separately. The values of these properties depend on the sequence-design method, structure predictor, sampling budget, filtering thresholds and database release. Circular validation may also arise when generators and evaluators share related architectures, training data or evolutionary information. Objective comparison therefore requires explicit redundancy filters, temporal splits, evaluator independence, candidate counts, and the reporting of unsuccessful experimental tests. Although PDB-Struct and Scaffold-Lab represent important steps towards unified assessment, community benchmarks that link computational outputs to experimentally measured properties are still necessary.

Addressing these limitations will require the integration of generative modelling with independent structure prediction, molecular simulation, uncertainty estimation and experimental feedback. Sequence, structure, dynamics and function may be approached within increasingly unified frameworks; nevertheless, their simultaneous optimisation remains a prospective objective. Future studies should prioritise reproducible benchmarks, accessible implementations, and the systematic reporting of both successful and unsuccessful designs. Closed-loop design–build–test–learn workflows have the potential to accelerate protein engineering; however, their reliability will require the establishment of objectives grounded in experimentation, the incorporation of uncertainty awareness in candidate selection, and the ongoing involvement of human oversight.

An additional limitation pertains to the developability of the protein. A generated sequence or structure may appear computationally designable, yet exhibit poor recombinant expression, limited solubility, aggregation, chemical instability, proteolytic sensitivity, immunogenicity or unsuitable oligomerisation. The properties in question are influenced by environmental and manufacturing conditions that are only weakly represented in most training datasets. Thus, practical protein engineering necessitates a comprehensive evaluation that extends beyond the conventional parameters of fold recovery and target activity. This evaluation should encompass a range of critical factors, including expression yield, monodispersity, long-term stability, and compatibility with downstream production or therapeutic use.

The practical comparison and application of diffusion models are constrained by two factors. Iterative denoising, repeated sequence design, structure prediction and post-generation filtering can require considerable computational resources, particularly in the context of long proteins or multicomponent systems. Furthermore, discrepancies in code accessibility, model weights, licences, hardware, sampling parameters and unpublished filtering procedures have the potential to impede independent replication. Therefore, benchmark studies should report the computational cost, software versions, random seeds and candidate-selection procedures alongside structural and functional metrics.

### 5.2. Toward Thermodynamics-Informed and Dynamics-Aware Protein Design

From a statistical–mechanical perspective, an equilibrium ensemble is defined under specified thermodynamic conditions and follows a Boltzmann-weighted distribution over molecular states. By contrast, the empirical distribution of structures deposited in databases and the distribution learned by a generative model are shaped by experimental selection, sampling limitations, dataset composition, and the training objective. Neither distribution is therefore equivalent, by construction, to an equilibrium ensemble. Recovering equilibrium populations requires calibrated statistical weights or free-energy differences, whereas kinetic interpretation additionally requires information on state connectivity and transition rates. Boltzmann generators and Markov state models illustrate the additional physical constraints and dynamical information required for such interpretations [45,46,47,48].

As diffusion models are capable of learning complex distributions of protein sequences and structures, the distributions represented by these models should not be assumed to correspond to physical equilibrium distributions. Statistical thermodynamics is an inherently probabilistic discipline; however, the empirical data distribution, the learned model distribution and the thermodynamic distribution of molecular conformations are, as a rule, distinct.

From the perspective of statistical thermodynamics, protein conformations are distributed across a rugged free-energy landscape, with their populations determined by sequence, temperature, solvent, molecular partners and other environmental conditions. Functional proteins often populate multiple metastable states, and biologically relevant conformations may be stabilised by ligands, cofactors, membranes or macromolecular interactions. Equilibrium populations and molecular stability are governed by free-energy differences that reflect enthalpic and entropic contributions. However, folding, conformational transitions, ligand association and enzymatic turnover also depend on activation barriers and kinetic pathways. Thermodynamic favourability, therefore, does not in itself establish that a molecular state is kinetically accessible or that a designed protein will fold, bind or function on biologically relevant timescales. The majority of contemporary diffusion models are optimised through the use of denoising, score-matching, or analogous generative objectives, as opposed to the employment of explicit molecular free-energy functions. Physical information may be inferred indirectly from the structural regularities embodied in the training data. However, the resulting scores are not calibrated thermodynamic quantities.

Thermodynamic interpretation is closely linked to conformational dynamics. The biological function of a system may be contingent upon the existence of multiple metastable states, the relative populations of these states, and the transition pathways that facilitate the connection between them. The generation of multiple structurally distinct conformations is therefore inadequate for the reconstruction of a physical ensemble. In order to support the thermodynamic interpretation, it is necessary to associate the sampled states with meaningful statistical weights. In order to support the kinetic interpretation, it is also necessary to characterise their connectivity and transition rates. Current ensemble-oriented diffusion models primarily address conformational diversity and do not yet consistently recover equilibrium populations or kinetic networks.

Future developments may combine learned generative priors with physical information at several stages of the design process. Generated structures can subsequently be filtered or refined using stereochemical validation, molecular mechanics, and molecular dynamics. Alternatively, differentiable energy terms or physically motivated restraints can be incorporated during the training or sampling phases. Conformational ensembles may also be reweighted using approximate free-energy estimates, while experimental measurements of stability, affinity or activity can be introduced through active-learning or design–build–test–learn cycles. These strategies differ substantially in terms of computational cost, accuracy and ease of integration, and should not be regarded as a single approach. Physics-based guidance has been demonstrated to enhance local geometry, interaction plausibility and the ranking of candidate structures. However, its impact on folding stability, binding affinity or catalytic activity is contingent upon the accuracy of the physical model and the adequacy of conformational sampling. Force fields remain approximate, solvent effects may be uncertain, and free-energy calculations are computationally demanding. Physics-informed generation should therefore be evaluated empirically rather than assumed to improve every design objective.

Consequently, thermodynamics-informed protein design should be formulated as condition-dependent inference rather than generic structural plausibility. Where possible, candidate structures should be associated with defined temperature, solvent, protonation, ligand and oligomeric conditions, and assessed through observables that can be related to these conditions. It cannot be assumed that a model trained on heterogeneous structural data represents a unique or universal protein free-energy landscape.

## 6. Conclusions

Diffusion-based protein design is comprised of several distinct methodological families, whose utility depends on the intended design objective. It has been demonstrated that RFdiffusion-derived methods are particularly well-suited to fixed-motif scaffolding, binder generation and, in their all-atom extensions, atom-level active-site and ligand-aware design. FrameDiff-based approaches provide a residue-frame formulation for unconditional backbone generation, structural inpainting and conditional sampling. By contrast, the Genie family emphasises diverse backbone exploration, multi-motif scaffolding and increasingly all-atom generation. Chroma is capable of supporting programmable combinations of geometric and semantic constraints. ProteinGenerator is able to couple sequence and structure generation. Mac-Diff targets learned conformational heterogeneity as opposed to reconstruction of a physically calibrated equilibrium ensemble. Across these categories, comparative outcomes are found to be strongly dependent on training datasets, sampling budgets, inverse-folding and structure-prediction pipelines, and evaluator choice. Consequently, stereochemical quality, structural self-consistency, novelty, diversity and task-specific constraint satisfaction should be reported separately and should not be interpreted as the direct evidence of folding free energy, kinetic accessibility or biological function. It is anticipated that future studies will yield the greatest insights when task-matched generative models are combined with condition-aware molecular modelling, uncertainty estimation, and experimental measurements of expression, stability, structure and function.

## Figures and Tables

**Table 1:** Notation used in the theoretical background.

Symbol	Description
$x_{0}$	Original protein representation (sequence, coordinates or latent variable)
$x_{t}$	Noisy sample at diffusion step *t*
*T*	Total number of diffusion steps
$\beta_{t}$	Variance (noise) schedule
$\alpha_{t}$	Single-step signal coefficient, $\alpha_{t}=1-\beta_{t}$
${\bar{\alpha }}_{t}$	Cumulative signal coefficient, ${\bar{\alpha }}_{t}=\prod_{s=1}^{t}\alpha_{s}$
$\epsilon_{\theta }$	Neural network predicting the added Gaussian noise
$\Sigma_{\theta }$	Reverse-process covariance
q(·)	Forward diffusion distribution
$p_{\theta }\left(\cdot \right)$	Learned reverse diffusion distribution
$\mu_{\theta }$	Predicted mean of the reverse transition
f(t)	Drift coefficient in the SDE
g(t)	Diffusion coefficient in the SDE
$\omega_{t}$	Standard Wiener process
$s_{\theta }$	Neural network estimating the score function
$\nabla_{x}\log p\left(x\right)$	Score function (gradient of log-density)

**Table 2:** Summary of representative diffusion-based protein structure methods according to molecular representation, design objective and representative use cases.

Category	Representative Models	Molecular Representation	Design Objective	Representative Applications
Backbone generation	RFdiffusion, FrameDiff, Genie	Backbone coordinates or residue-local frames	Unconditional de novo backbone generation	Generation of novel monomeric folds, exploration of backbone topology, and production of backbone libraries for subsequent inverse folding
Motif-guided generation	RFdiffusion, Genie2, FrameDiPT, TDS	Backbone coordinates combined with fixed motifs or partial structures	Motif scaffolding, structural inpainting and conditional generation	Scaffolding of catalytic or binding motifs, peptide- and protein-binder generation, multi-motif scaffolding, and reconstruction of missing structural regions
Programmable generation	Chroma	Coordinates combined with user-defined constraints or energy functions	User-specified conditional design	Symmetric protein generation, control of global shape, preservation of substructures, and semantic or functional-class conditioning
All-atom generation	RFdiffusionAA, RFdiffusion2, RFdiffusion3, Genie3	Backbone, side-chain and non-protein atomic coordinates	Atom-level and ligand-aware design	Design around small molecules, cofactors and nucleic acids; specification of hydrogen bonds, catalytic geometries and ligand-contact networks
Interaction prediction	AlphaFold3	Heterogeneous biomolecular systems	Conditional complex-structure prediction	Prediction and evaluation of protein–protein, protein–ligand, protein–nucleic-acid and modified biomolecular complexes
Sequence-structure co-design	ProteinGenerator	Coupled amino-acid sequence and three-dimensional structure	Joint sequence and structure generation	Multistate design, functional protein generation and simultaneous optimisation of sequence-structure compatibility
Ensemble generation	Mac-Diff	Protein sequence and trajectory-derived conformational states	Conditional conformational ensemble generation	Sampling of alternative conformational states and exploration of conformational heterogeneity

**Table 3:** Hierarchical interpretation of metrics and frameworks used to evaluate generated protein structures.

Metric or Framework	Validation Layer	What It Measures	What It Does Not Establish
MolProbity	Local stereochemistry	Steric clashes, Ramachandran outliers, side-chain rotamers and selected bond-geometric deviations	Global fold stability, correct oligomerisation, favourable solvation, binding affinity or biological function
RMSD	Structural similarity	Mean geometric deviation between aligned atomic coordinates	Predictor confidence, fold novelty, thermodynamic stability or functional equivalence; values depend on alignment and protein size
TM-score	Global fold similarity	Length-normalised similarity between two protein structures	Local stereochemical correctness, sequence compatibility, energetic favourability or biological activity
pLDDT	Predictor confidence	Per-residue confidence assigned by a structure-prediction model	Experimentally measured accuracy, thermodynamic stability, expression, foldability or function
Sequence recovery	Sequence-design consistency	Fraction of native or target amino-acid identities recovered by an inverse-folding model	Structural novelty, correct folding of a new sequence or functional activity
Structural self-consistency	Predicted designability	Whether a sequence designed for a generated backbone is predicted to recover that backbone	Independent experimental foldability; results depend on the selected sequence-design and structure-prediction models
Novelty	Distributional comparison	Dissimilarity of a generated structure to entries in a reference structural database	Designability, stability or function; estimates depend on database release and structural-search protocol
Diversity	Distributional coverage	Number or proportion of distinct structural clusters among generated candidates	Structural quality, physical realism or experimental success
PDB-Struct	Structure-based design benchmarking	Sequence recovery, refoldability and correlation between model scores and experimentally annotated mutational stability data	Direct stability or foldability of an individual newly generated protein
Scaffold-Lab	Generative-model benchmarking	Designability, novelty, diversity, structural quality, motif recovery and computational efficiency under a common protocol	Wet-lab expression, stability, affinity, catalytic activity or biological function
Molecular mechanics, MD and free-energy calculations	Physical and thermodynamic assessment	Energetic compatibility, conformational relaxation and, under defined conditions, approximate relative free energies or populations	Guaranteed experimental stability or function; results depend on force fields, solvent models and sampling convergence
Experimental expression and biophysical characterisation	Experimental tractability and structure	Expression yield, solubility, monodispersity, secondary structure and, where available, atomic or near-atomic structure	Intended molecular function unless a task-specific functional assay is also performed
Binding, catalytic or cellular assays	Functional validation	Affinity, specificity, catalytic activity or cellular response under the tested conditions	General activity under other conditions, long-term stability, developability or absence of off-target effects

**Figure A1:**
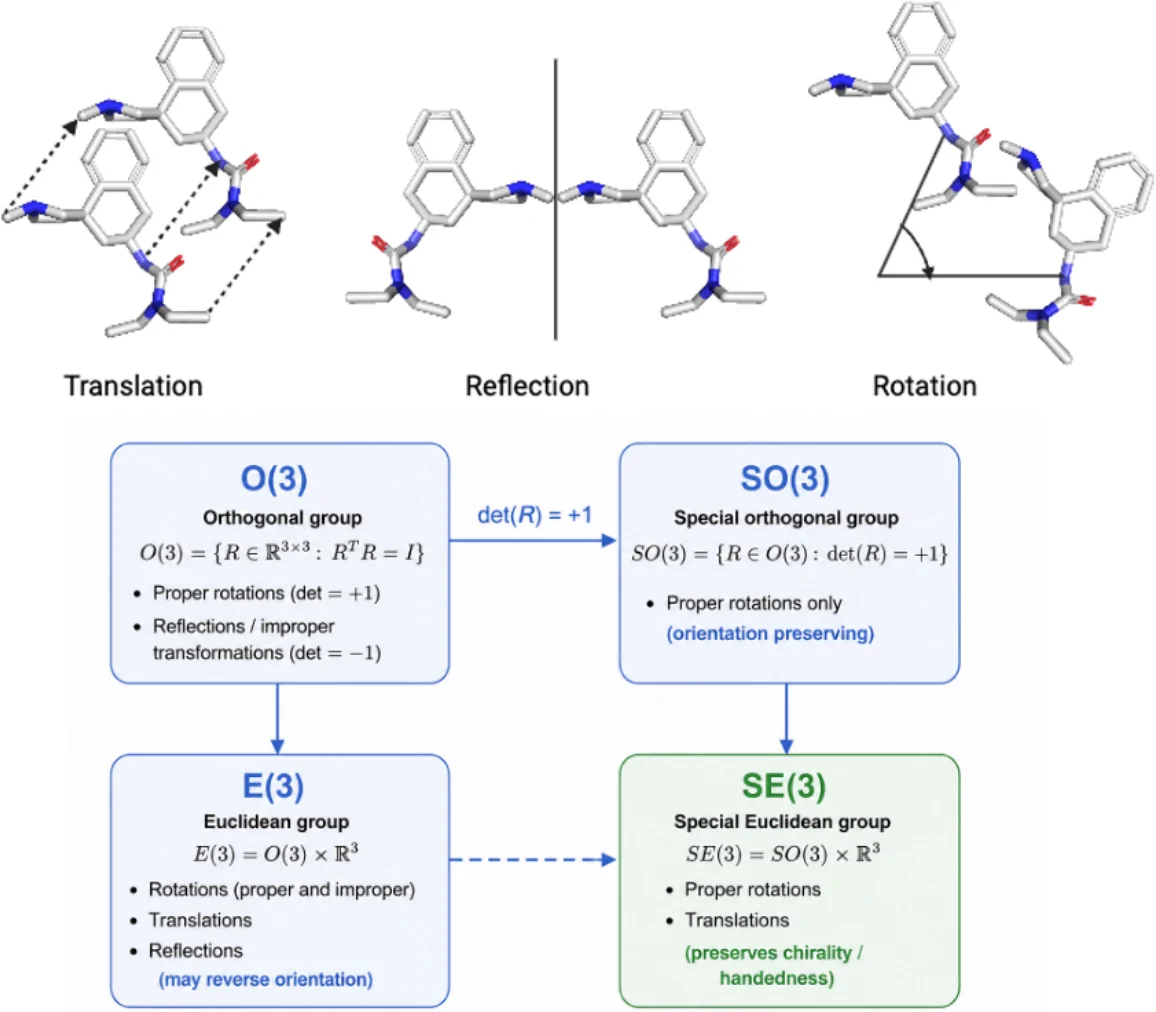
Information about E(3) group. Top subplot: illustration of translation, reflection and rotation. Bottom subplot: relationship among several general groups.

## Data Availability

No new experimental data were generated in this study, as this article is a review of previously published research. The publications, datasets, benchmark frameworks, and software resources discussed in this review are publicly available through the original sources cited in the manuscript. Relevant resources include the Protein Data Bank (PDB) and the publicly available repositories associated with the methods and benchmark frameworks reviewed herein. Readers are referred to the corresponding original publications and repositories for dataset versions, access conditions, software licenses, and implementation details.

## Abbreviations

The following abbreviations are used in this manuscript:

DDPM	Denoising Diffusion Probabilistic Models
AF2	AlphaFold2
SGM	Score-Based Generative Models
AF3	AlphaFold3
VAEs	Variational Autoencoders
PLM	Protein Language Model
MSE	Mean Squared Error
SDE	Stochastic Differential Equation
GNN	Graph Neural Network
ODE	Ordinary Differential Equation
CGD	Context-Guided Diffusion
RFDiffusion	RoseTTAFold Diffusion
RFAA	RoseTTAFold All-Atom
IPA	Invariant Point Attention
GVP	Geometric Vector Perception
VFN	Vector Field Networks
FrameDiPT	FrameDiff inPainTing
TDS	Twisted Diffusion Sampler
DFM	Discrete Flow Model
pLDDT	Predicted Local Distance Difference Test
SubDiff	Subgraph Latent Diffusion Model
PAE	Predicted Alignment Error
RMSD	Root Mean Square Deviation
GL(n)	General Linear Group
SE(3)	Special Euclidean 3D Group

## Appendix A. Model Overview

### Model List

Models mentioned in this review have been implemented as open-source tools. We list their tasks, inputs, outputs, training datasets, dataset sizes and code links in Table A1. This table may help users with their research problems and help developers to further improve them.

**Table A1:** Overview of the representative diffusion-based protein design models discussed in this review. For each model, the table summarises its primary design task, molecular input and output representations, training dataset, dataset size (when available), code availability (accessed on 27 July 2026), and corresponding reference.

Paper	Input	Output	Dataset	Data Size	Code	Ref
RFDiffusion	structures	structures	PDB	-	https://github.com/RosettaCommons/RFdiffusion	[13]
RFAA	sequence	structures	PDB	121,800	https://github.com/baker-laboratory/rf_diffusion_all_atom	[8]
RFDiffusion2	structure	structures	PDB	-	https://github.com/RosettaCommons/RFdiffusion2/	[31]
RFDiffusion3	structure	structures	PDB	-	https://github.com/RosettaCommons/foundry/blob/production/models/rfd3	[32]
Protein Generator	sequence and structure	sequence and structure	PDB	-	https://github.com/RosettaCommons/protein_generator	[23]
FrameDiff	structures	structures	PDB	20,312 backbones	https://github.com/jasonkyuyim/se3_diffusion	[14]
FrameDiPT	structures	structures; all-atom	RCSB; PDB	9K clusters	https://github.com/instadeepai/FrameDiPT	[33]
TDS	structures	structures	-	-	https://github.com/blt2114/twisted_diffusion_sampler	[34]
SMCDiff	motif	scaffolds	PDB	4269	https://github.com/blt2114/ProtDiff_SMCDiff	[49]
VFN-Diff	structures	structures	PDB	-	https://github.com/aim-uofa/VFN	[35]
Genie	structures	structures	SCOPe	195,214	https://github.com/aqlaboratory/genie	[15]
Genie2	structures	structures	PDB; AFDB	588,570 structures	https://github.com/aqlaboratory/genie2	[36]
Genie3	structures	structures	PDB; AFDB	587,479 AFDB structures + 135,745 Pinder complexes	https://github.com/aqlaboratory/genie3	[37]
Mac-Diff	protein sequence	protein conformational ensembles	PDB; GPCRmd; ATLAS	619,045 PDB structures + 1674 MD trajectories	https://github.com/Paulie-ai/Mac-Diff	[38]
Chroma	sequence	structures	PDB, UniProt, PFAM	28,819 structures	https://github.com/generatebio/chroma	[16]
AlphaFold3	sequence; SMILES	structures	PDB 2021	41,000,000 structures	https://github.com/google-deepmind/alphafold3	[7]

## Appendix B. SE(3) Group

SE(3) equivariance has been repeatedly mentioned in this review and plays an important role in the structure generation of proteins, small molecules, and protein ligands. Here, we give its definition, properties, and relationship with the protein framework.

### Appendix B.1. Definition of SO(3) and SE(3)

In mathematics, a rigid body can be abstracted into 3-dimensional geometric coordinates. A rotation of a set of three-dimensional coordinates can be represented by a 3×3 rotation matrix. For rotation only, this can be written as

$$\left(\begin{array}{ccc} x_{1} & y_{1} & z_{1}\\ \vdots & \vdots & \vdots \\ x_{n} & y_{n} & z_{n} \end{array}\right)\times \underset{\mathrm{denoted}\;\mathrm{as}\;A}{\underset{︸}{\left(\begin{array}{ccc} a_{1} & b_{1} & c_{1}\\ a_{2} & b_{2} & c_{2}\\ a_{3} & b_{3} & c_{3} \end{array}\right)}}=\left(\begin{array}{ccc} x_{1}^{\prime } & y_{1}^{\prime } & z_{1}^{\prime }\\ \vdots & \vdots & \vdots \\ x_{n}^{\prime } & y_{n}^{\prime } & z_{n}^{\prime } \end{array}\right)$$

**Definition A1.** 
*(Representation) An n-dimensional real representation of a group G is a map $\rho :G\to R^{n\times n}$, assigning to each g∈G an invertible matrix ρ(g), and satisfying*

ρ(e)=1,ρ(gh)=ρ(g)ρ(h),∀g,h∈G.

According to Definition A1, group elements can be represented by matrices through a matrix representation. In the following, we introduce several matrix groups that are commonly used to describe rigid-body transformations.

The general linear group GL(n) is the group of all invertible n×n matrices.

The orthogonal group in three dimensions is defined as $O\left(3\right)=\left\{R\in R^{3\times 3}:R^{T}R=I\right\}$. It contains both proper rotations and improper transformations such as reflections. The special orthogonal group

SO(3)={R∈O(3):det(R)=+1}

contains proper rotations only.

The Euclidean group $E\left(3\right)=O\left(3\right)⋉R^{3}$ contains rotations, translations, and reflections, whereas the special Euclidean group $SE\left(3\right)=SO\left(3\right)⋉R^{3}$ contains proper rotations and translations only.

Because protein structures are chiral, reflections require particular care in molecular modelling. SE(3)-equivariant formulations are therefore commonly used when preserving molecular handedness is required. E(3)-equivariant formulations may still be useful in appropriate settings, but their relationship to chirality should be stated explicitly. Inner product and distance on SE(3) are as follows:

$${\langle A_{1},A_{2}\rangle }_{SE(3)}={\langle \gamma_{1},\gamma_{2}\rangle }_{SO(3)}+{\langle s_{1},s_{2}\rangle }_{R^{3}}$$

$$d_{SE(3)}\left(A_{1},A_{2}\right)=\sqrt{d_{SO(3)}{\left(\gamma_{1},\gamma_{2}\right)}^{2}+d_{R^{3}}{\left(s_{1},s_{2}\right)}^{2}}.$$

SE(3) satisfies the following four axioms:

- The set is closed under the binary operation. A,B∈SE(3)⇒AB∈SE(3).
- The binary operation is associative. A,B,C∈SE(3)⇒(AB)C=A(BC).
- ∃I∈SE(3), s.t., ∀A∈SE(3), AI=A.
- ∀A∈SE(3), $\exists A^{-1}\in SE\left(3\right)$, $AA^{-1}=I$.

SE(3) is a six-dimensional Lie group and can be locally parameterised by three rotational and three translational degrees of freedom. In other words, SE(3) is a differentiable manifold, i.e., a Lie group.

For a protein frame, the atomic coordinates can be defined as $\left[N_{n},C_{n},{\left(C_{\alpha }\right)}_{n}\right]=\left[T_{n}\right]\cdot \left[N^{*},C^{*},C_{\alpha }^{*}\right]$, *n* is the index of residue, $r_{n}=$ Gram–Schmidt($v_{1},v_{2}$), $x_{n}=C_{\alpha }\in R^{3}$, $T_{n}=\left(r_{n},x_{n}\right)$ is a member of the special Euclidean group SE(3).

### Appendix B.2. Relationship Between SE(3) and E(3)

The Euclidean group E(3) consists of all rigid-body transformations in three-dimensional space, including translations, proper rotations, and reflections. Therefore, E(3) preserves distances but not necessarily orientation.

The special Euclidean group SE(3) is the subgroup of E(3) consisting of proper rotations and translations only. Since proper rotations have determinant +1, SE(3) preserves both distances and molecular handedness (orientation). The relationship between SE(3) and E(3) (see Figure A1) is as follows: SE(3) is a subgroup of E(3) that includes only translation and rotation, while E(3) includes the motions of SE(3) as well as reflective motions. In other words, SE(3) is a subset of E(3) that remains oriented.

E(3)-equivariant neural networks can be computationally more efficient and have been shown to perform as well as or better than SE(3)-equivariant networks for the modelling of quantum chemical properties and dynamic systems [50].
